# Low skeletal muscle mass index and all-cause mortality risk in adults: A systematic review and meta-analysis of prospective cohort studies

**DOI:** 10.1371/journal.pone.0286745

**Published:** 2023-06-07

**Authors:** Yahai Wang, Donglin Luo, Jiahao Liu, Yu Song, Binggang Jiang, Haichao Jiang

**Affiliations:** 1 College of Arts and Physical Education, Nanchang Normal College of Applied Technology, Nanchang, Jiangxi, China; 2 Faculty of Health Service, Naval Medical University, Shanghai, China; National Institute of Biomedical Innovation Health and Nutrition, JAPAN

## Abstract

**Objective:**

The relationship between low skeletal muscle mass index (SMI) and all-cause mortality risk in the general adults remains unclear. Our study was conducted to examine and quantify the associations between low SMI and all-cause mortality risks.

**Methods:**

PubMed, Web of Science, and Cochrane Library for primary data sources and references to relevant publications retrieved until 1 April 2023. A random-effect model, subgroup analyses, meta-regression, sensitivity analysis, and publication bias were conducted using STATA 16.0.

**Results:**

Sixteen prospective studies were included in the meta-analysis of low SMI and the risk of all-cause mortality. A total of 11696 deaths were ascertained among 81358 participants during the 3 to 14.4 years follow-up. The pooled RR of all-cause mortality risk was 1.57 (95% CI, 1.25 to 1.96, P < 0.001) across the lowest to the normal muscle mass category. The results of meta-regression showed that BMI (P = 0.086) might be sources of heterogeneity between studies. Subgroup analysis showed that low SMI was significantly associated with an increased risk of all-cause mortality in studies with a body mass index (BMI) between 18.5 to 25 (1.34, 95% CI, 1.24–1.45, P<0.001), 25 to 30 (1.91, 95% CI, 1.16–3.15, P = 0.011), and over 30 (2.58, 95% CI, 1.20–5.54 P = 0.015).

**Conclusions:**

Low SMI was significantly associated with the increased risk of all-cause mortality, and the risk of all-cause mortality associated with low SMI was higher in adults with a higher BMI. Low SMI Prevention and treatment might be significant for reducing mortality risk and promoting healthy longevity.

## Introduction

Skeletal muscles were the largest organs in the human body, accounting for roughly 20–40% of total body weight in women and 30–50% in males and functioning as the largest body protein reservoir [1], which was crucial to human health [2]. There was evidence that skeletal muscle mass declined with age [3]. At the age of 30, inactive individuals began to experience a steady loss in skeletal muscle mass, and the rate of deterioration of skeletal muscle mass got more pronounced after the age of 60 [4, 5]. This might not only lead to sarcopenia, a multifactorial syndrome defined as a disease [6], but also increase the incidence rate and mortality [7]. Sarcopenia was a sickness defined by a loss of skeletal muscle mass and strength that could raise the risk of physical disability, chronic diseases such as diabetes and cardiovascular disease [8], and it was highly linked to mortality in older adults [9]. It is not surprising that the age-related reduction in muscle mass has become a major global health concern, given the growing global population of older people.

Indeed, clinical research has identified low skeletal muscle mass as an independent predictor of mortality in chronic diseases such as chronic liver disease [10], cardiovascular diseases [11], and end-stage renal failure [12], and poor skeletal muscle mass is predictive of negative surgical outcomes in elderly individuals with cancer [13, 14]. The links between skeletal muscle mass decline and all-cause mortality in the general population are now equivocal despite the accumulation of evidence. The results of studies examining the relationships between muscle mass or sarcopenia and mortality are inconclusive. Recently, several studies have found that assessing muscle strength, as opposed to muscle mass, was more important [15, 16]. Others indicate that muscle mass is predictive of adverse outcomes and mortality [17–19], which may have been due to the wide variety of muscle mass testing technologies and different muscle mass indexes.

To the best of our knowledge, no available systematic review and meta-analysis were found on the relationship of low skeletal muscle mass index (SMI) with all-cause mortality in the general population. Previous relevant studies often focused on the elderly [20] or diseased populations [21–23]. It is commonly recognized that skeletal muscle and healthy aging are tightly associated. Thus, it is essential to identify the risk of all-cause mortality attributable to low SMI among the general population. In this study, we aimed to conduct a systematic review and meta-analysis of prospective cohort studies to investigate the association between low SMI and all-cause mortality risk among adults.

## Methods

This study was registered on PROSPERO (ID: CRD42022343325). The Preferred Reporting Items for Systematic Reviews and Meta-Analyses (PRISMA) 2020 guidelines were used to conduct this meta-analysis [24].

### Search strategy

The PubMed, Web of Science, and Cochrane Library were scoured for relevant publications until 1 April 2023. S1 Table outlines the search approach in detail. We also ran a thorough search for pertinent article reference lists.

### Study selection

In the first search, two authors (Yahai Wang and Donglin Luo) independently assessed titles and abstracts before reviewing the full-text of all eligible publications. The third author (Jiahao Liu) mediated the disagreements in order to establish a consensus. We considered prospective cohort studies that assessed the association between skeletal mass index and mortality in the general population. These were the inclusion criteria for this review: (1) The study design was a prospective cohort study; (2) the exposure of interest was skeletal muscle mass index (SMI); (3) the outcome was all-cause mortality; and (4) the researchers reported relative risk (RR), hazard ratio (HR), or odds ratio (OR) of outcome risk along with corresponding 95% confidence intervals (CIs). In the meantime, the exclusion criteria were as follows: (1) participants were not recruited from an overall healthy population; and (2) reviews, randomized controlled trials (RCTs), case-control studies, retrospective cohort studies, nonhuman studies, non-English studies, and letters lacking sufficient data were excluded. In the event of numerous reports from the same study, only those with the longest follow-up and the biggest sample size were considered.

### Outcomes

The outcome was all-cause mortality.

### Data extraction

The process of data extraction was carried out by two trained researchers. The data from each qualifying study were extracted onto a standard form, which included the surname of the first author, publication year, study design, study location, sample size (total sample/number of deaths), mean age, follow-up years, body mass index (BMI) of participants, the method used for assessment of SMI (e.g., dual-energy X-ray absorptiometry (DXA), bioimpedance analysis (BIA), anthropometry, computed tomography (CT) and magnetic resonance imaging (MRI)), predictors reported for SMI (equals to ASM/height^2^, or ALM/height^2^, or SMM/height^2^) and matching effect size of comparison categories together with 95% confidence intervals (CIs) and variables in the fully adjusted model. If several predictors of muscle mass were reported, the most popular and well-known predictor would be examined. If studies provided data independently by gender, they would be examined as two distinct reports.

### Quality assessment

Two qualified researchers independently assessed the quality of all included studies using the Newcastle-Ottawa Scale (NOS) in terms of selection (4 stars), comparability (2 stars), and outcomes (3 stars) [25]. Higher study scores reflect a higher quality of study. We assessed 0–3, 4–6, and 7–9 NOS scores to be of low, medium, and high quality, respectively. The Grading of Recommendations, Assessment, Development, and Evaluation (GRADE) method was applied in order to evaluate the quality of the evidence that was presented for the outcomes [26]. According to the GRADE guideline, study design determines the baseline quality of the evidence, e.g., observational studies were initially assigned a ranking of low, and other factors could downgrade or upgrade the quality of evidence. Disagreements were resolved via dialogue with the third reviewer (Haichao Jiang).

### Statistical analysis

For the purpose of comparing the all-cause mortality risks associated with having the low SMI to those associated with having normal SMI (reference), a random-effects model was utilized to pool risk estimates with 95 percent confidence intervals, to produce more conservative results than a model with a fixed-effect. Version 5.1.0 of the Cochrane Handbook for Systematic Reviews of Interventions, HR was roughly equivalent to RR [27]. In the meantime, an OR was converted to an RR using the following formula: RR = OR/[(1-P0)+(P0*OR)], where P0 represents the reference group’s mortality rate [28]. We measured the heterogeneity between studies using the Q test and the I^2^-statistic [29]. The presence of significant heterogeneity was indicated by a P-value less than 0.1 in the Q test or an I^2^ greater than 50%.

Subgroup analyses and meta-regression were performed to explore potential sources of between-study heterogeneities from age at baseline, gender, mean BMI at baseline, location of study (specific country for meta-regression), duration of follow-up, number of participants, method used for assessment of SMI, study quality, and adjustment for confounders (not performed for meta-regression). Additionally, a meta-regression model was also performed with continuous variables, except for method of exposure assessment, and country of study. A P-value of less than 0.1 was considered statistically significant for meta-regression analysis.

A leave-one-out meta-analysis (LOOM) was undertaken as a sensitivity analysis, i.e., deleting one piece of research at a time to evaluate the robustness of the primary results and the influence of each report on the effect or heterogeneity. Publication bias was evaluated by funnel plots and Egger’s regression test. The presence of publication bias was indicated by a P-value smaller than 0.1 [30]. In cases of publishing bias, the trim and fill method was applied [31].

To prevent input mistakes, we ran data analyses using STATA version 16.0 (Stata Corp, College Station, TX, USA) with double data input. Unless otherwise noted, a P-value of less than 0.05 was regarded as statistically significant.

## Results

### Literature search

**Fig 1:**
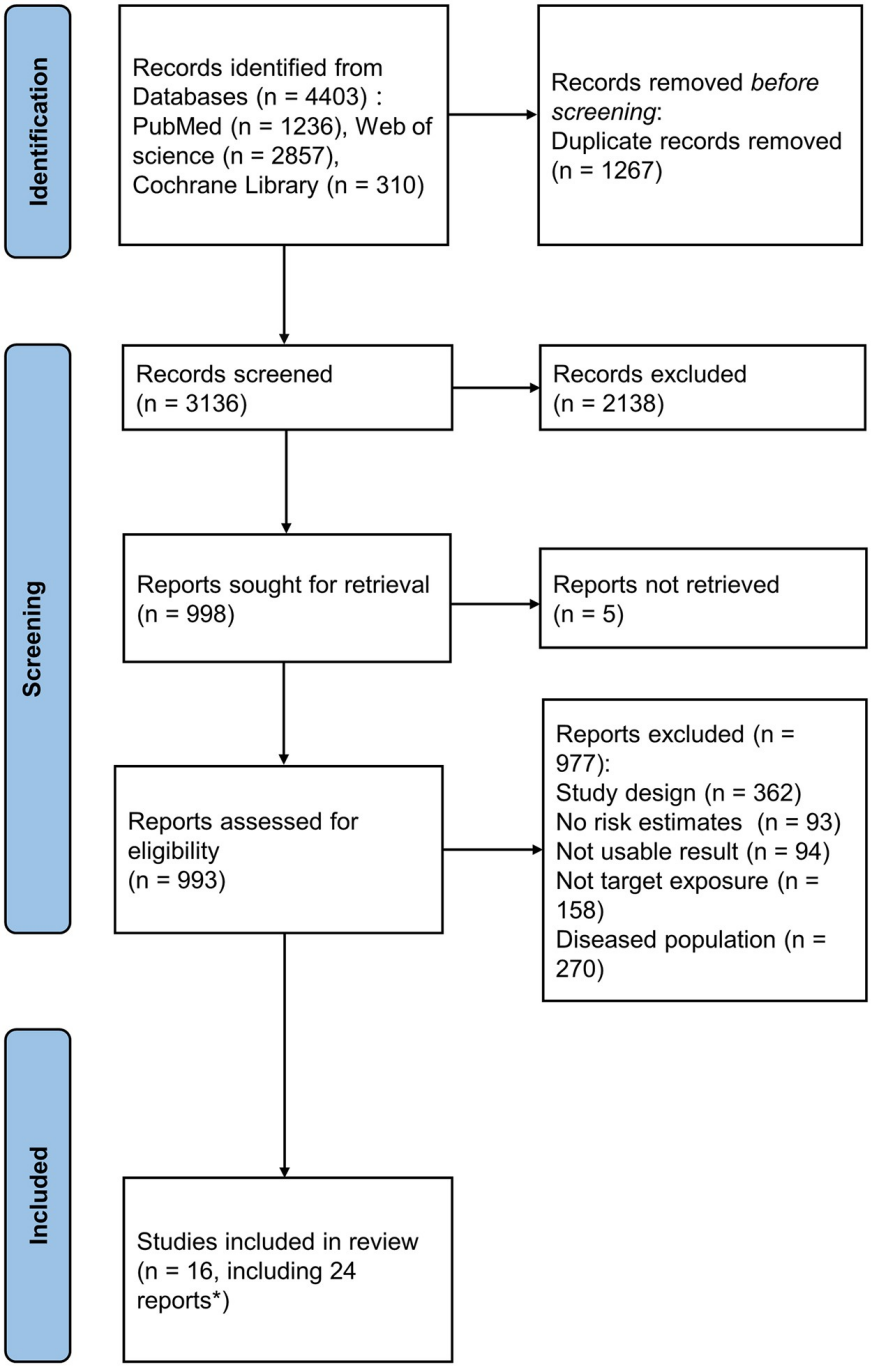
Flow chart of study selection.

The flowchart depicting the study selection procedure is shown in Fig 1. Initial retrieval yielded 4403 articles, but after removing duplicates and screening titles and abstracts, only 993 articles remained for full-text review. An additional 977 articles were removed for the following reasons: 362 Study design was inconsistent; participants in 270 studies were not healthy; 158 articles had improper measurements, 94 articles did not report assessable target outcomes; and 93 articles lacked adequate data for quantitative analysis. In the final meta-analysis, 16 studies [32–47] involving 81358 participants were included.

### Characteristics of included studies

Sixteen articles with twenty-four reports reported skeletal muscle mass index-related mortality risk effect sizes [32–47]. Table 1 displayed a summary and details of the sixteen articles included in the review. The studies included 81358 participants, of whom 11696 passed away. The number of deaths was not reported in a single study [36]. The range of sample sizes was from 287 to 44060. The participants’ average age ranged from 43,9 to 93,5 years. The average duration of follow-up varied between 3 and 14.4 years. To detect SMI, ten studies utilized DXA [32, 34, 37, 38, 40, 42–46] five studies utilized BIA [33, 35, 36, 39, 41], and two studies utilized Anthropometry [43, 47].

**Table 1:** Characteristics of included studies for all-cause mortality (16 trials).

Author, Year	Country	Study name *	N (deaths)	Mean age at baseline (years)	Follow-up years	Exposure assessment	Muscle wasting categories	Corresponding relative risk (95% CI)
Abramowitz, 2018	USA	NHANES, 1999–2004	11687 (1819)	46.4	9.3	DXA	ASMI, kg/m^2^: Low ASMI (BMI 18.5–25) Low ASMI (BMI 25–30) Low ASMI (BMI 30–40)	All-cause mortality: 1.28 (1.07, 1.52) 1.52 (1.22, 1.89) 2.58 (1.20, 5.53)
Buchman, 2021	USA	Rush Memory and Aging Project	1466 (579)	81.3	5.5	BIA	SMI (per SD decrease), kg/m^2^	All-cause mortality: 1.15 (1.04, 1.28)
Cawthon, 2021	USA	MrOS Study 2000–2014	5849 (1630)	73.6	14.4	DXA	ALM/height^2^, kg/cm^2^: Quintile 3 (7.70–8.13) Quintile 2 (7.21–7.7 Quintile 1 (<7.21)	All-cause mortality: 1 1.04 (0.88, 1.22) 1.38 (1.18, 1.60)
Chuang, 2014	China	Elderly NAHSIT 1999–2000	Male:785 (298) Female:727 (208)	Male: 72.0 Female: 71.6	9.19	BIA	SMMI, kg/m^2^: Normal Male<11.45 Female<8.51	All-cause mortality: 1 1.28 (0.96, 1.70) 1.58 (1.12, 2.22)
Costanzo, 2020	Italy	InCHIANTI Study 2001–2010	535(/)	77.0	10	BIA	SMMI, kg/m^2^: Normal Low muscle mass	All-cause mortality: 1 2.69 (1.04, 6.94)
de Santana, 2019	Brazil	SPAH Study	Male:323 (65) Female:516 (67)	Male: 72.9 Female: 73.4	4.06 ± 1.07	DXA	ALM/height^2^, kg/m^2^: Male: Normal Low muscle mass Female: Normal Low muscle mass	All-cause mortality: 1 5.21(1.94, 7.75) 1 9.97 (7.92, 11.01)
Kim, 2014	Korea	KLoSHA Study	Male: 284 (40) Female: 272 (19)	Male: 74.8 Female: 73.3	6	DXA	ASM/hight^2^, kg/m^2^: Male: ≤7.09 Female: ≤5.27	All-cause mortality: 3.12 (1.08, 9.02) 1.24 (0.32, 4.77)
Moon, 2016	Korea	KLoSHA Study	Male: 285 (40) Female: 275 (21)	Male: 74.3 Female: 73.2	6	DXA	Male: ASM/height^2^ (20% decrease) ASM/BMI (20% decrease) Female: ASM/height^2^ (20% decrease) ASM/BMI (20% decrease)	All-cause mortality: 1.53 (0.72, 3.24) 3.02 (1.40, 6.47) 0.96 (0.30, 3.06) 0.76 (0.21, 2.77)
Kruse, 2020	USA	CHS Study (1989–2005)	Male: 2459 Female: 3295 Total cases:1174	73.0	7	BIA	SMI, kg/m^2^: Male: ≥10.76 8.51–10.75 ≤ 8.50 Female: ≥6.76 5.76–6.75 ≤5.75	All-cause mortality: 1 1.06 (0.91,1.22) 1.26 (1.05,1.50) 1 1.02 (0.94,1.11) 1.04 (0.90,1.21)
Nakamura, 2020	Japan	Hisayama Study	2968 (87)	74.1	4.3	BIA	Low muscle mass (SMI, kg/m^2^: Male< 7.0 Female< 5.7)	All-cause mortality: 1.10 (0.67, 1.80)
Oh, 2020	Korea	KNHANES 2007–2014	44060 (1682)	43.9	2–9.5	DXA	SMI > lowest tertile SMI < lowest tertile	All-cause mortality: 1 1.53 (1.33, 1.76)
Sanada, 2018	USA	Kuakini Honolulu Heart Program	2309 (2210)	77.6	11.7	Anthropometry	SMI, kg/m^2^: ≥7.77 <7.77	All-cause mortality: 1 1.26 (1.15, 1.38)
Sim, 2019	Australia	Perth Longitudinal Study of Aging in Women	903 (263)	79.9	9.5	DXA	ALM/height^2^ (per SD decrease), kg/m^2^	All-cause mortality: 1.00 (0.89, 1.14)
Sobestiansky, 2019	Sweden	ULSAM	287 (60)	86.6	3	DXA	Low SMI, kg/m^2^	All-cause mortality: 1.56 (1.22, 2.00)
Spahillari, 2016	USA	CHS Study (two DXA-scanned study sites) (1989–2013)	1335 (1047)	76.2	12	DXA	SMI, per 1 kg/m^2^ decrease	All-cause mortality: 1.12 (1.03, 1.23)
Wang, 2019	China	PLAD	Male: 238 (132) Female: 500 (255)	Male: 93.2 Female: 93.7	4	Anthropometry	SMI, kg/m^2^: Male: <5.58 Female: <3.38	All-cause mortality: 0.82 (0.45, 1.47) 1.54 (1.10, 2.16)

Abbreviations: ALM, appendicular lean mass; ASMI, appendicular skeletal mass index; BIA, bioelectrical impedance; BMI, body mass index; DXA, dual x-ray absorptiometry; q, quartile; Q, quintile; SMI, skeletal mass index; SMMI, skeletal muscle mass index.

*, NHANES, National Health and Nutrition Examination Survey; MrOS, Osteoporotic Fractures in Men; InCHIANTI, “Invecchiare in Chianti”; Elderly NAHSIT, Elderly Nutrient and Health Survey in Taiwan; SPAH, São Paulo Ageing & Health; KLoSHA, Korean Longitudinal Study on Health and Aging; KNHANES, Korea National Health and Nutrition Examination Survey; ULSAM, Uppsala Longitudinal Study of Adult Men; PLAD, The Project of Longevity and Aging in Dujiangyan; CHS, Cardiovascular Health.

### Study quality

NOS was used to evaluate the quality of the study, and the scores are shown in Table 2. According to the NOS score, fifteen of the studies were of high quality, one was of medium quality, and none were of low quality. The average study quality score for all-cause mortality was 7.7. The evidence level of the results can be seen in S2 Table.

**Table 2:** Study quality of studies included in the analysis assessed by the Newcastle Ottawa Scale.

Author, publication year	Selection	Comparability	Outcome	Total score
Abramowitz, 2018	****	*	***	8
Buchman, 2021	****	**	**	8
Cawthon, 2021	***	**	***	8
Chuang, 2014	***	**	**	7
Costanzo, 2020	***	**	***	8
de Santana, 2019	****	**	**	8
Kim, 2014	****	**	**	8
Kruse, 2020	****	**	**	8
Moon, 2016	****	**	**	8
Nakamura, 2020	****	**	**	8
Oh, 2020	****	**	**	8
Sanada, 2018	***	**	**	7
Sim, 2019	***	-	***	6
Sobestiansky, 2019	***	**	**	7
Spahillari, 2016	***	**	***	8
Wang, 2019	****	**	**	8

Selection: 1) Representativeness of the exposed cohort; 2) Selection of the non-exposed cohort; 3) Ascertainment of exposure; 4) Demonstration that outcome of interest was not present at start of study; Comparability: 1a) study controls for age (the most important factor); 1b) study controls for any additional factor; Outcome: 1) Assessment of outcome; 2) Was follow-up long enough (≥ 5 years) for outcomes to occur; 3) Adequacy of follow up of cohorts (≥ 80%).

### Low skeletal muscle mass index and mortality risk

**Fig 2:**
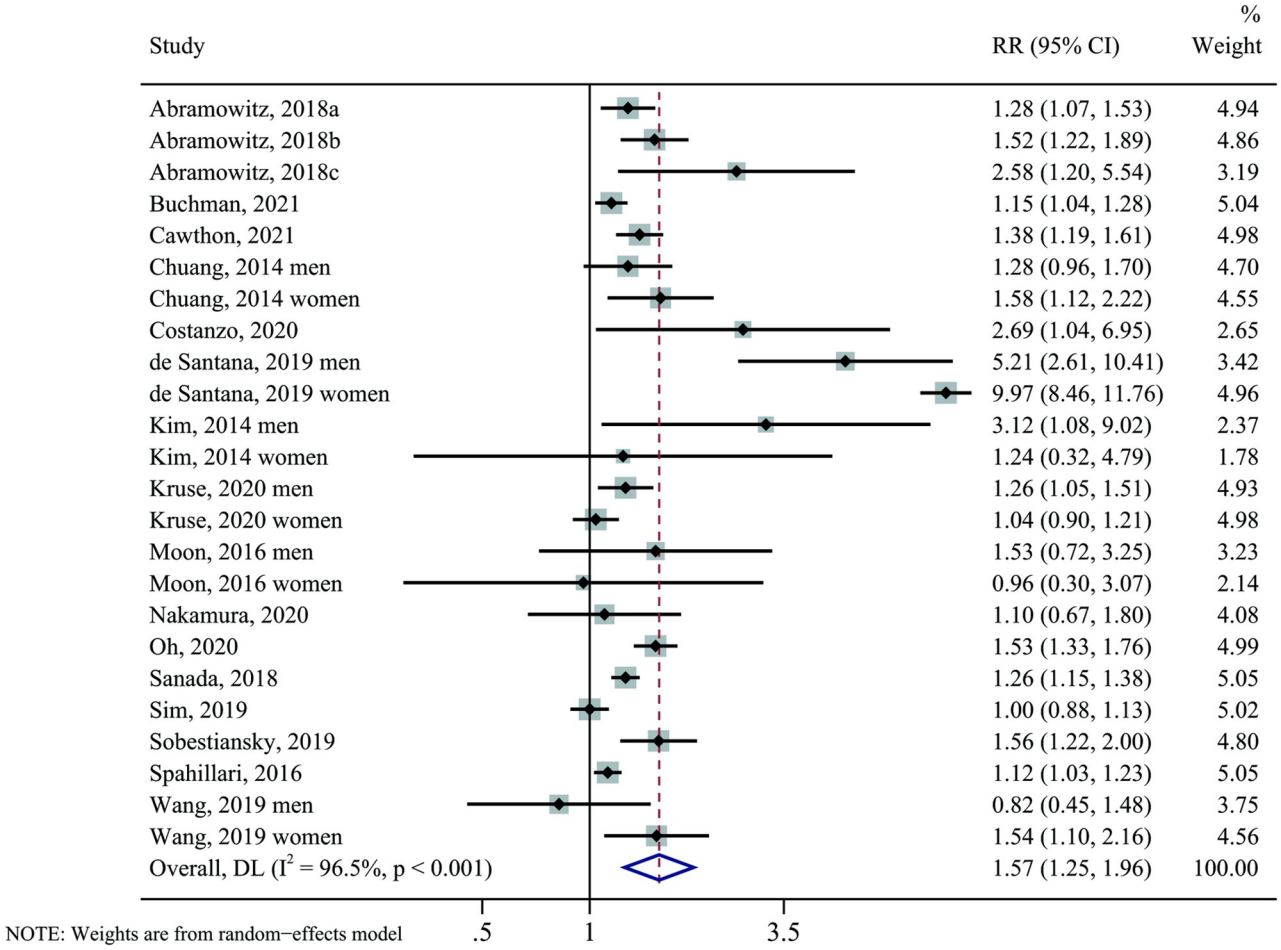
The forest plot of low skeletal muscle mass index (lowest *vs*. normal category of muscle mass) and the risk of all-cause mortality by pooling data from 16 studies. RR indicates relative risk, CI indicates confidence intervals.

In total, 16 studies with 24 reports were included in the analysis of low SMI and the risk of all-cause mortality. The pooled RR of all-cause mortality risk was 1.57 (95% CI, 1.25 to 1.96, P < 0.001) across the lowest to normal muscle mass category, indicating a significant positive association between low SMI and all-cause mortality risk. High heterogeneity was observed among studies (I^2^ = 96.5%, P < 0.001) (Table 3 and Fig 2).

**Table 3:** Subgroup analysis of low skeletal muscle mass index and risk of all-cause mortality.

Variables	n	RR (95% CI)		P^1^	Heterogeneity		meta-regression
					I^2^ (%)	P^2^	P^3^
**All-cause mortality**	**24**	**1.57 (1.25 to 1.96)**		**<0.001**	**96.5**	**<0.001**	
**Age at baseline**							0.611
**≤ 45 years**	**2**	**1.41 (1.19 to 1.68)**		**<0.001**	**58.8**	0.119	
**45~65 years**	**2**	**1.74 (1.11 to 2.72)**		**0.016**	**41.3**	0.192	
**≥ 65 years**	**20**	**1.56 (1.20 to 2.04)**		**0.001**	**97.1**	**<0.001**	
**Gender**							0.819
**Male**	**9**	**1.41 (1.21 to 1.64)**		**<0.001**	**66.2**	**0.003**	
**Female**	**7**	1.67 (0.72 to 3.91)		0.233	**98.9**	**<0.001**	
**Mixed**	**8**	**1.32 (1.16 to 1.51)**		**<0.001**	**72.2**	**0.001**	
**BMI at baseline, kg/m** ^**2**^							**0.086**
**Underweight (BMI<18.5)**	**2**	1.18 (0.64 to 2.17)		0.595	**69.6**	0.070	
**Normal (18.5≤BMI<25)**	**10**	**1.34 (1.24 to 1.45)**		**<0.001**	9.2	0.358	
**Overweight (25≤BMI<30)**	**8**	**1.91 (1.16 to 3.15)**		**0.011**	**98.9**	**<0.001**	
**Obesity (BMI≥30)**	**1**	**2.58 (1.20 to 5.54)**		**0.015**	**-**	**-**	
**Location**							**0.021**
**Asia**	**8**	**1.41 (1.24to 1.64)**		**<0.001**	**17.4**	0.293	
**Europe**	**3**	1.40 (0.95 to 2.06)		0.089	**78.4**	**0.010**	
**USA**	**8**	**1.21 (1.12 to 1.31)**		**<0.001**	**57.7**	**0.021**	
**Anthropometry**	**3**	3.71 (0.61to 22.52)		0.154	**99.6**	**<0.001**	
**Follow-up years**							0.217
**< 5**	**6**	2.21 (0.84 to 5.82)		0.110	**98.1**	**<0.001**	
**5~10**	**14**	**1.28 (1.15 to 1.44)**		**<0.001**	**67.0**	**<0.001**	
**≥ 10**	**4**	**1.25 (1.11 to 1.42)**		**<0.001**	**66.7**	**<0.029**	
**No. participants**							0.822
**< 5000**	**17**	**1.65 (1.19 to 2.29)**		**0.003**	**97.5**	**<0.001**	
**≥ 5000**	**7**	**1.34 (1.18 to 1.52)**		**<0.001**	**69.2**	**0.003**	
**Exposure assessment**							
**DXA**	**14**	**1.86 (1.26 to 2.74)**		**0.002**	**97.9**	**<0.001**	0.182
**BIA**	**7**	**1.20 (1.07 to 1.33)**		**0.001**	**37.2**	0.144	
**Others**	**3**	**1.27 (1.02 to 1.57)**		**0.031**	**40.7**	0.185	
**Adjustment for confounder**							
**Physical activity**	**Yes**	**11**	**1.89 (1.11 to 3.22)**	**0.018**	**98.3**	**<0.001**	
	**No**	**13**	**1.30 (1.16 to 1.45)**	**<0.001**	**73.9**	**<0.001**	
**Hypertension**	**Yes**	**9**	**1.93 (1.14 to 3.27)**	**0.014**	**98.7**	**<0.001**	
	**No**	**15**	**1.35 (1.21 to 1.51)**	**<0.001**	**66.8**	**<0.001**	
**Diabetes**	**Yes**	**11**	**1.81 (1.12 to 2.93)**	**0.016**	**98.3**	**<0.001**	
	**No**	**13**	**1.35 (1.20 to 1.52)**	**<0.001**	**71.2**	**<0.001**	
**Stroke**	**Yes**	**4**	1.19 (0.95 to 1.49)	0.125	**51.3**	0.104	
	**No**	**20**	**1.60 (1.24 to 2.06)**	**<0.001**	**97.1**	**<0.001**	
**Smoking/alcohol**	**Yes**	**16**	**1.58 (1.16 to 2.15)**	**0.004**	**97.5**	**<0.001**	
	**No**	**8**	**1.31 (1.10 to 1.55)**	**0.002**	**69.8**	**<0.001**	

P^1^value for RR; P^2^ value for heterogeneity between studies; P^3^ value for meta-regression; significant p-values are highlighted in bold prints.

Abbreviations: BMI, body mass index.

## Meta-regression and subgroup analysis

**Fig 3:**
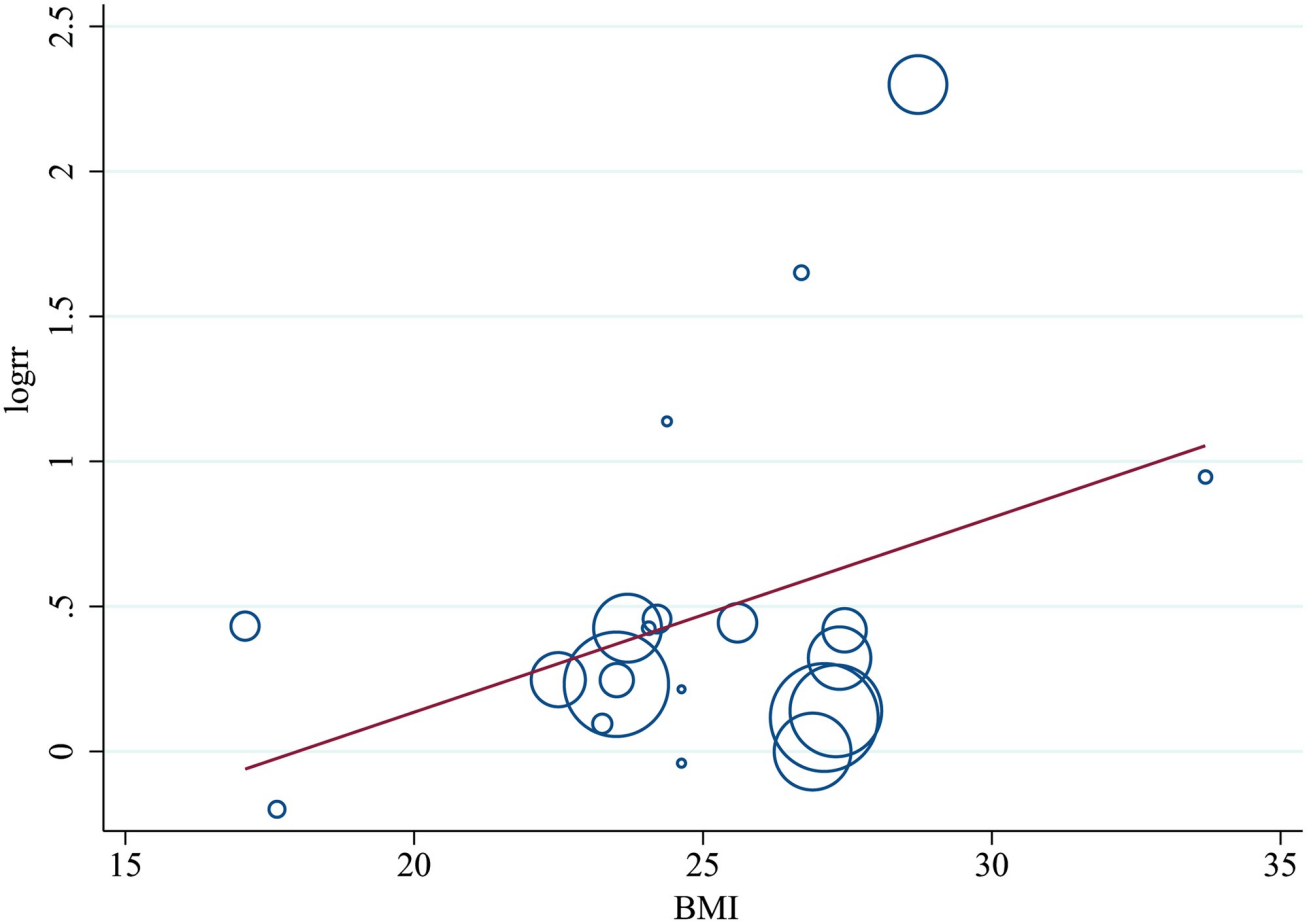
Meta-regression model for the effect of low skeletal muscle mass index on the risk of all-cause mortality adjusted by body mass index of study. RR indicates relative risk.

The results of subgroup analysis and meta-regression were shown in Table 3. We stratified by age, gender, BMI, country, follow-up time, number of participants, and measurement methods. Stratified by BMI (P = 0.086) and country (P = 0.021), heterogeneity was found between studies. In addition, the risk of all-cause mortality associated with low SMI was higher in adults with a higher BMI (Fig 3).

According to the findings of the subgroup analysis, low SMI was significantly associated with an increase in all-cause mortality in people aged ≤ 45, 45 to 65, and ≥ 65, and the pooled RRs were 1.41 (95% CI, 1.19–1.68, P<0.001), 1.74 (95% CI, 1.11–2.72, P = 0.016), 1.56 (95% CI, 1.20–2.04, P = 0.001), respectively. low SMI was significantly associated with an increased risk of all-cause mortality in male studies and mixed male and female studies, the pooled RRs were 1.41 (95% CI, 1.21–1.64, P<0.001) and 1.32 (95% CI, 1.16–1.51, P<0.001). Additionally, low SMI was significantly associated with an increased risk of all-cause mortality in studies with a body mass index (BMI) between 18.5 to 25 (1.34, 95% CI, 1.24–1.45, P<0.001), 25 to 30 (1.91, 95% CI, 1.16–3.15, P = 0.011), and over 30 (2.58, 95% CI, 1.20–5.54 P = 0.015). Geographically, low SMI was significantly associated with increased risk of all-cause mortality in Asian (1.41, 95% CI, 1.24–1.64, P<0.001) and American (1.21, 95% CI, 1.12–1.31, P<0.001) countries. Low SMI was significantly associated with an increased risk of all-cause mortality in studies with a follow-up time of 5 to 10 years (1.28, 95% CI, 1.15–1.44, P<0.001) and more than 10 years (1.25, 95% CI, 1.11–1.42, P<0.001). According to the testing technologies, low SMI was significantly associated with an increased risk of all-cause mortality in studies using BIA (1.20, 95% CI, 1.07–1.33, P = 0.001), DXA (1.86, 95% CI, 1.26–2.74, P = 0.002), and anthropometry (1.27, 95% CI, 1.02–1.57, P = 0.031). Therefore, gender, BMI, geography, duration of follow-up, and testing technologies might be sources of heterogeneity in the study.

### Sensitivity analysis

**Fig 4:**
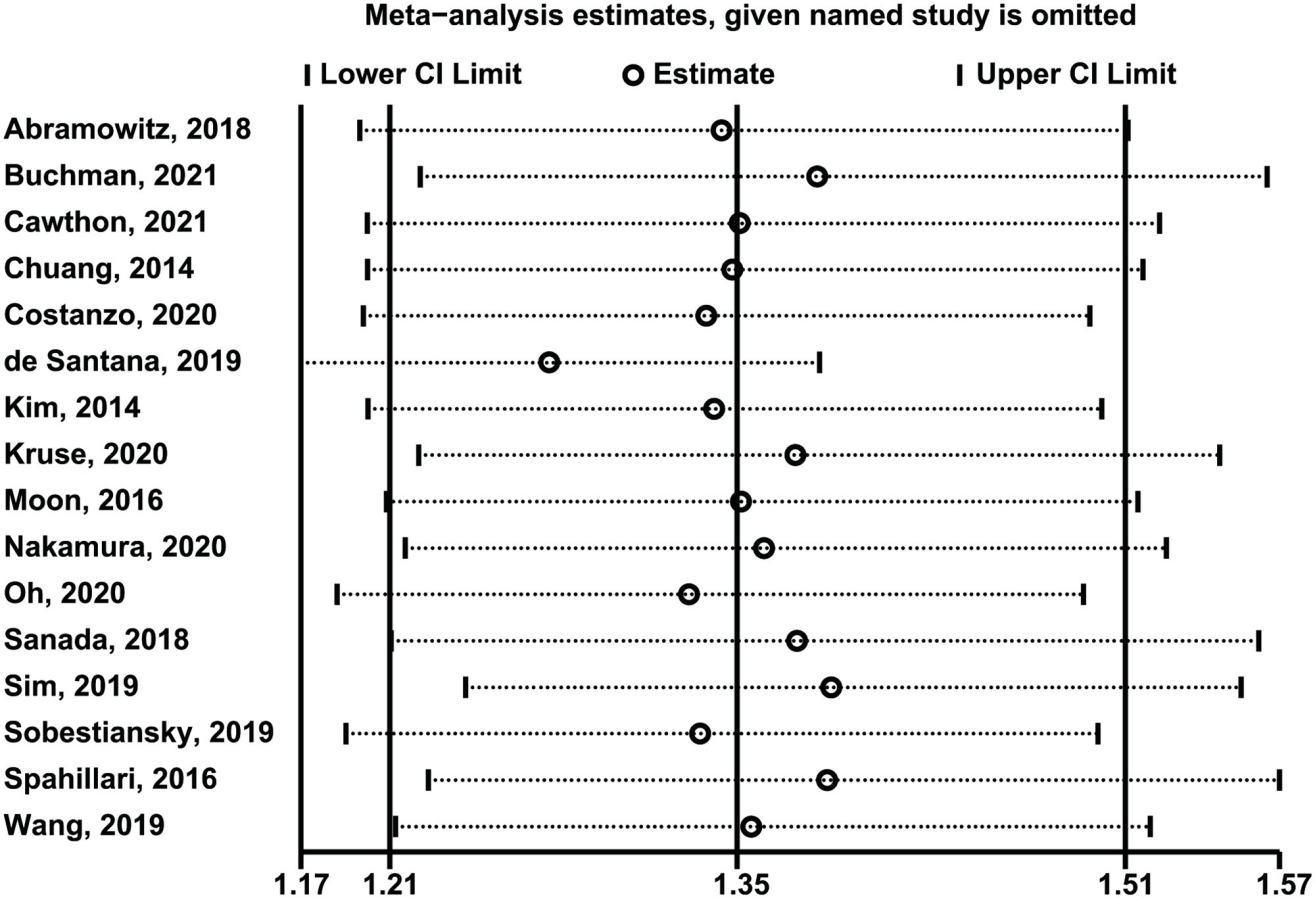
Sensitive analysis of low skeletal muscle mass index (lowest vs. normal category of muscle mass) and all-cause mortality. RR indicates relative risk, CI indicates confidence interval.

Concerning the robustness of overall effect sizes, we conducted LOOM analysis for sensitivity. The results of sensitivity analysis showed that after excluding any single study at a time, low skeletal muscle mass index was still significantly associated with the increase in all-cause mortality risk, indicating that our meta-analysis results were robust (Fig 4) (S3 Table).

### Publication bias

**Fig 5:**
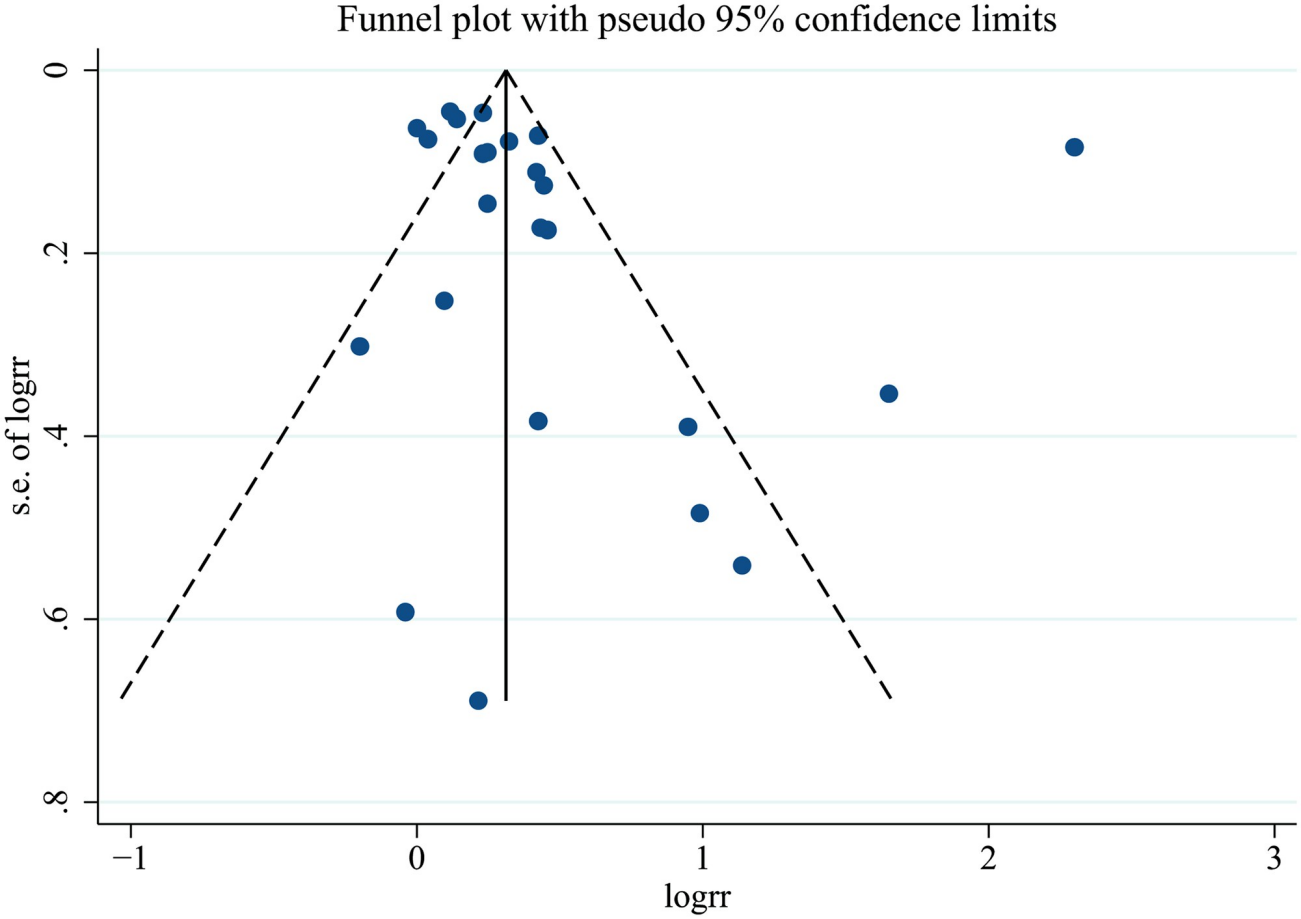
Funnel plot of low skeletal muscle mass index and the risk of all-cause mortality. RR indicates relative risk.

The Egger’s test and funnel plot indicated no significant publication bias in the primary analysis for all-cause mortality (P = 0.364) (Fig 5).

## Discussion

This is a meta-analysis based on 16 prospective cohort studies, and the results showed that the pooled RR of all-cause mortality risk was 1.57 (95% CI, 1.25 to 1.96, P<0.001) across the lowest to the normal SMI, indicating a significant positive association between low SMI and all-cause mortality risk, and the risk of all-cause mortality associated with low SMI was higher in adults with a higher BMI. In addition, meta-regression and subgroup analysis showed that gender, BMI, country, location, duration of follow-up, and assessment methods might be sources of heterogeneity in the study.

Our results were consistent with the results of a previous meta-analysis [20] based on 9 cohort studies, which showed that low skeletal muscle mass was significantly associated with an increased risk of all-cause mortality. However, our research was very different in study design. The previous meta-analysis study only retrieved two databases, and the study objects only included the elderly over 65 years old. The most critical thing was that they calculated the standardized mean difference in skeletal muscle mass index between the dead and the living by comparing them with the survivors. Muscle loss or decreased muscle mass was significantly connected with an increased risk of all-cause mortality in diseased individuals [22, 23]. This suggested that low SMI was strongly related with an increased risk of all-cause mortality, regardless of the health status of individuals. Thus, increased attention should be paid to this public health issue among the general population to minimize the burden of death to the greatest extent.

This meta-analysis found that low SMI was significantly associated with an increase in all-cause mortality in people aged ≤ 45 years old, 45 to 65 years old, and ≥ 65 years old. Because there were too few samples in the two subgroups (≤ 45 years old and between 45 and 65 years old), the exact correlation between low SMI and all-cause mortality could not be obtained, which needed to be verified by more cohort studies in the later stage. Subgroup analysis of subgroups older than 65 years old showed that low SMI increased the risk of all-cause mortality by 56%, which might be related to the faster decline of muscle mass [4] and the decline of their own physical function [48] in the elderly. Therefore, we should pay more attention to the serious phenomenon of muscle loss in the elderly and prevent it in advance.

Meta-regression results were consistent with subgroup analyses, indicating that the risk of all-cause mortality associated with low SMI was higher in adults with a higher BMI. Interestingly, a cohort study [49] based on 3.6 million adults also found similar results, BMI had J-shaped associations with all-cause mortality. Despite the fact that everyone is aware that obesity is damaging to health. Numerous studies [50–52] demonstrated that obesity increased mortality, but the optimal BMI range was still unknown. The reason may be that the causal relationship and possible mechanisms underlying between BMI and age, sex, and other specific associations have not been found [53].

The results of meta regression and subgroup analysis indicated that studies in different regions might be the source of heterogeneity in this meta-analysis, which might be attributed to diverse ethnic groups in different countries and varied instruments and equipment used to measure SMI.

As we all know, there were many measurements of muscle mass, including skeletal mass index (SMI), skeletal muscle mass index (SMMI), appendicular lean mass (ALM), appendicular muscle mass (AMM), appendicular skeletal mass index (ASMI), body muscle mass index (BMMI), fat-free mass index (FFMI), and mid-arm muscle circumference (MAMC). Considering that there may be errors between different measurement methods, resulting in great heterogeneity between studies, our study included only prospective cohort studies that reported skeletal muscle quality by SMI (equals to ASM/height^2^, or ALM/height^2^, or SMM/height^2^). This meta-analysis found that the subgroups using DXA(1.86, 95% CI, 1.26–2.74, P = 0.002) and BIA (1.20, 95% CI, 1.07–1.33, P = 0.001) to measure SMI showed that low SMI was significantly associated with an increased risk of all-cause mortality. Currently, DXA and BIA are widely used to measure muscle mass [54, 55], but both have a major drawback in that the measurement findings of different manufacturers of instruments are inconsistent [56–59]. However, clinical researchers have been trying to find ways to optimize the loopholes of both, attempting to pinpoint the actual cut-off point [60]. In addition, from an economic and practical standpoint, the BIA method of measuring muscle mass might be superior to DXA [61], which also explained to some extent why the heterogeneity of BIA (I^2^ = 37.2, P = 0.144) was smaller.

In addition, we discovered that the risk of all-cause mortality was lower in studies with a follow-up of more than 10 years compared to studies with a follow-up of 5 to 10 years. A meta-analysis of the connection between sarcopenia and all-cause mortality risk discovered a decreased all-cause mortality risk in trials lasting five years or more [62]. One possible reason might be that the subjects of the study with a follow-up of less than 5 years were older. In the studies we included, the average age of the participants was 81.8 years [37, 41, 45, 47] for follow-up periods of less than 5 years, 68.0 years [32, 33, 35, 38–40, 42, 44] for follow-up periods of 5 to 10 years, and 76.1 years [34, 36, 43, 46] for follow-up periods of more than 10 years. Although the average age of participants with a follow-up duration of 5 to 10 years was older than that of those with a follow-up period of more than 10 years, the average age of those with a follow-up period of less than 5 years was the oldest.

This study had some limitations. Despite the fact that we unified the indicators for evaluating skeletal muscle mass index (SMI), significant heterogeneity was still observed. Through subgroup analysis and meta-regression, we found the possible potential sources of heterogeneity. Nevertheless, the study was still limited by its small sample size. Last, studies reported low SMI diagnosed by different criteria were included in this present meta-analysis, thus the differences in different assessment criteria are inevitable.

## Conclusions

Low SMI was significantly associated with the increased risk of all-cause mortality, and the risk of all-cause mortality associated with low SMI was higher in adults with a higher BMI. Low SMI prevention and treatment might be significant for reducing mortality risk and promoting healthy longevity.

## Supporting information

S1 File PRISMA 2020 checklist. (DOCX)

S1 Table Search strategy. (DOCX)

S2 Table Grades of Recommendation, Assessment, Development and Evaluation (GRADE) quality of evidence. (DOCX)

S3 Table Leave-one-out meta-analysis of low skeletal muscle mass index and risk of all-cause mortality. (DOCX)
