# Supplementary material for: Low skeletal muscle mass index and all-cause mortality risk in adults: A systematic review and meta-analysis of prospective cohort studies
Source: PLoS One. 2023 Jun 7;18(6):e0286745. doi: 10.1371/journal.pone.0286745 (PMC10246806; doi:10.1371/journal.pone.0286745)
Supplement: S1 Table — (DOCX) [file pone.0286745.s002.docx]

**S1 Table. Search strategy.**

| Example search strategy in PubMed. |
| --- |
| ((“skeletal muscle mass” [Title/Abstract]) OR (“lean body weight” [Title/Abstract]) OR (“skeletal muscle mass index” [Title/Abstract]) OR (“fat-free mass” [Title/Abstract]) OR (“lean mass” [Title/Abstract]) OR (“lean body mass” [Title/Abstract]) OR (“lean tissue mass” [Title/Abstract])) AND ((death [Title/Abstract]) OR (“death rate” [Title/Abstract]) OR (mortality [Title/Abstract]) OR (mortalities [Title/Abstract]) OR (fatal [Title/Abstract]) OR (survival [Title/Abstract]) OR (deceased [Title/Abstract]) OR (“survival rate” [Title/Abstract])) AND ((cohort studies [Mesh]) OR cohort OR prospective OR follow-up) NOT ((“comment”[Publication Type]) OR (“letter”[Publication Type])) |
| Example search strategy in Web of science and Cochrane library. |
| ((“skeletal muscle mass”) OR (“Skeletal muscle”) OR (“Low skeletal muscle”) OR (“Low skeletal muscle mass”) OR (“skeletal muscle loss”) OR (“fat-free mass”) OR (“lean mass”) OR (“lean body mass”) OR (“lean tissue mass”)) AND ((death) OR (“death rate”) OR (mortality) OR (mortalities) OR (fatal) OR (survival) OR (deceased) OR (“survival rate”)) AND (“cohort studies” OR cohort OR prospective OR follow-up) NOT ((“comment”[Publication Type]) OR (“letter”[Publication Type])) |
