# Supplementary material for: Low skeletal muscle mass index and all-cause mortality risk in adults: A systematic review and meta-analysis of prospective cohort studies
Source: PLoS One. 2023 Jun 7;18(6):e0286745. doi: 10.1371/journal.pone.0286745 (PMC10246806; doi:10.1371/journal.pone.0286745)
Supplement: S2 Table — (DOCX) [file pone.0286745.s003.docx]

**S2 Table. Grades of Recommendation, Assessment, Development and Evaluation (GRADE) quality of evidence.**

| **Outcomes** | **Risk of**  **bias** | **Inconsistency** | **Indirectness** | **Imprecision** | **Publication**  **bias** | **Effect**  **size** | **Plausible**  **residual**  **confounding** | **Dose-response**  **gradient** | **GRADE**  **rating** |
| --- | --- | --- | --- | --- | --- | --- | --- | --- | --- |
| **All-cause mortality** | 0 | -1^a^ | 0 | 0 | 0 | +1^b^ | 0 | 0 | Low |

a. There is signiﬁcant and unexplained variability in results from different trials; b. Large effect size.
