# Supplementary material for: Low skeletal muscle mass index and all-cause mortality risk in adults: A systematic review and meta-analysis of prospective cohort studies
Source: PLoS One. 2023 Jun 7;18(6):e0286745. doi: 10.1371/journal.pone.0286745 (PMC10246806; doi:10.1371/journal.pone.0286745)
Supplement: S3 Table — (DOCX) [file pone.0286745.s004.docx]

**S3 Table. Leave-one-out meta-analysis of Low skeletal muscle mass index and risk of all-cause mortality.**

| **Study** **omitted** | **RR (95% CI)** | **P^1^** | **Heterogeneity** | |
| --- | --- | --- | --- | --- |
|  |  |  | **I^2^ (%)** | **P^2^** |
| Abramowitz, 2018 | **1.34 (1.19 to 1.51)** | **<0.001** | **81.0** | **<0.001** |
| Buchman, 2021 | **1.38 (1.22 to 1.56)** | **<0.001** | **81.0** | **<0.001** |
| Cawthon, 2021 | **1.35 (1.20 to 1.52)** | **<0.001** | **81.0** | **<0.001** |
| Chuang, 2014 | **1.35 (1.20 to 1.51)** | **<0.001** | **81.2** | **<0.001** |
| Costanzo,2020 | **1.33 (1.19 to 1.49)** | **<0.001** | **80.9** | **<0.001** |
| Santana,2019 | **1.27 (1.17 to 1.38)** | **<0.001** | **65.1** | **<0.001** |
| Kim, 2014 | **1.34 (1.20 to 1.50)** | **<0.001** | **81.2** | **<0.001** |
| Kruse, 2020 | **1.37 (1.22 to 1.54)** | **<0.001** | **81.4** | **<0.001** |
| Moon, 2016 | **1.35 (1.20 to 1.51)** | **<0.001** | **81.5** | **<0.001** |
| Nakamura, 2020 | **1.36 (1.21 to 1.52)** | **<0.001** | **81.5** | **<0.001** |
| Oh, 2020 | **1.33 (1.18 to 1.49)** | **<0.001** | **78.8** | **<0.001** |
| Sanada, 2018 | **1.37 (1.21 to 1.56)** | **<0.001** | **81.5** | **<0.001** |
| Sim, 2019 | **1.39 (1.24 to 1.55)** | **<0.001** | **78.0** | **<0.001** |
| Sobestiansky, 2019 | **1.33 (1.19 to 1.50)** | **<0.001** | **80.6** | **<0.001** |
| Spahillari, 2016 | **1.38 (1.22 to 1.57)** | **<0.001** | **80.1** | **<0.001** |
| Wang, 2019 **1.35 (1.21 to 1.52) <0.001 81.5 <0.001** | | | | |

P^1^ value for RR; P^2^ value for heterogeneity between studies; significant p-values are highlighted in bold prints.
